# Distributed Denial of Services (DDoS) attack detection in SDN using Optimizer-equipped CNN-MLP

**DOI:** 10.1371/journal.pone.0312425

**Published:** 2025-01-27

**Authors:** Sajid Mehmood, Rashid Amin, Jamal Mustafa, Mudassar Hussain, Faisal S. Alsubaei, Muhammad D. Zakaria

**Affiliations:** 1 Department of Computer Science and IT, University of Chakwal, Chakwal, Pakistan; 2 Department of Computer Science, University of Chakwal, Chakwal, Pakistan; 3 Faculty of Informatics and Computing, University Sultan Zainal Abidin, Besut, Terengganu, Malaysia; 4 Department of Computer Science and Creative Technologies, Global College of Engineering and Technology, Muscat, Oman; 5 Department of Cybersecurity, College of Computer Science and Engineering, University of Jeddah, Jeddah, Saudi Arabia; Study World College of Engineering, INDIA

## Abstract

Software-Defined Networks (SDN) provides more control and network operation over a network infrastructure as an emerging and revolutionary paradigm in networking. Operating the many network applications and preserving the network services and functions, the SDN controller is regarded as the operating system of the SDN-based network architecture. The SDN has several security problems because of its intricate design, even with all its amazing features. Denial-of-service (DoS) attacks continuously impact users and Internet service providers (ISPs). Because of its centralized design, distributed denial of service (DDoS) attacks on SDN are frequent and may have a widespread effect on the network, particularly at the control layer. We propose to implement both MLP (Multilayer Perceptron) and CNN (Convolutional Neural Networks) based on conventional methods to detect the Denial of Services (DDoS) attack. These models have got a complex optimizer installed on them to decrease the false positive or DDoS case detection efficiency. We use the SHAP feature selection technique to improve the detection procedure. By assisting in the identification of which features are most essential to spot the incidents, the approach aids in the process of enhancing precision and flammability. Fine-tuning the hyperparameters with the help of Bayesian optimization to obtain the best model performance is another important thing that we do in our model. Two datasets, InSDN and CICDDoS-2019, are utilized to assess the effectiveness of the proposed method, 99.95% for the true positive (TP) of the CICDDoS-2019 dataset and 99.98% for the InSDN dataset, the results show that the model is highly accurate.

## 1. Introduction

Online services are susceptible to Distributed Denial of Service (DDoS) attacks, which are designed to make the service unavailable through the massive amount of traffic sent to the target. Using bots that leverage compromised devices to perform targeted coordinated attacks, botnets become the weapon of choice for malicious actors [1]. That’s why these challenges are considered sophisticated: it is difficult to eradicate them [2–4]. The cybersecurity panorama is getting more and more complicated since, recently, the trends show an increase in the number of sophisticated DDoS techniques, such as the amplification attacks that take advantage of the vulnerabilities in the Internet of Things (IoT) devices [5–7]. Centralized designs of the Software-Defined Networking (SDN) architectures had made it a perfect target of choice for the hackers who used their structure exploits to launch powerful DDoS attacks [7].

DDoS detection is more advanced when using various ML methodologies. The first thing Is the feature selection, that is an approach to find relevant features in datasets, which improves the model’s ability to recognize DDoS attacks [8, 9]. Only relevant features are used in feature selection, which decreases dimensionality and removes superfluous features, thus guaranteeing deployment of the detection system with maximum efficiency and effectiveness. Next, deep learning (DL) is another conversation when it comes to ML applications in the detection of DDoS. Several-layer neural network arrangements are used in deep learning to discover intricate patterns and representations from data [10–12]. The sophisticated network traffic analysis the model performs enables it to identify the patterns of stealthy attacks notwithstanding the mass of data it can process [13]. In addition, the methods for enhancing the detection systems can also help optimize the DDoS better. The strategies utilized for combating this issue include hybrid deep learning models, which are the combination of different neural network topologies to utilize their diversity in dealing with particular DDoS attack problems [14]. The accuracy of DDoS detecting instruments is augmented by optimizing model selection techniques that combine methods such as stacking, bagging, and boosting [15–17]. This paragraph aims to show the importance of involving advanced machine learning methods like feature selection and deep learning, and also various optimization strategies in the process of DDoS attack detection and consequently mitigating the resulting threats. The primary area of cognitive computing is machine learning (ML), which is a field that strives to create intelligent systems that can learn from data and make well-informed forecasts and decisions on their own without the need for explicit programming [18–20]. We can point out that ML machine learning is mainly implemented in the following forms: semi-supervised, unsupervised, supervised, and reinforcement learning differ in how they learn conclusions from data. With machine learning (ML) as a core element of Distributed Denial of Service (DDoS) attack detection, it is easy to understand this capability because of its inherent ability to learn and keep improving itself over time [21–23].

The ML’s ability to identify and reduce DDoS attacks has been the main focus of recent years. Under the same scenario, machine learning is gaining popularity because its learning aspects can evolve and develop over time by applying different methods. An example of such approach is shrinking datasets to remove potential error sources by locating the crucial features within datasets required to train models to learn and separate malicious traffic from the normal ones. The main approach is optimisation methods to enhance the effectiveness of the ML-based DDoS detection systems [24, 25]. Three algorithms are used for machine learning: optimization and parameterization, model architecture, and fine-tuning of hyperparameters. The optimization techniques guarantee that the machine learning models are calibrated to face the complexities of DDoS detection. As a result, precision and effectiveness are improved.

Deep learning is the key to strengthening security protocols, which is especially important due to the variety of threats in the digital sphere. Deep learning has reshaped cyber security by introducing methods to detect malware, inspections, intrusions, network traffic, spam emails, and other problems. Such methods as DCNN, RNN, SAE, and BERT powered by sophisticated techniques have become indispensable tools that enhance electronic information security [26]. Security systems can now detect fake data injections, validate keystrokes, detect anomalies, and effectively manage insider threats because of deep learning algorithms. The level of intrusion detection systems in IoT networks is far more advanced than before because of the deployment of deep learning and security cyber solutions. These approaches use deep learning models to ensure the safety of IoT devices and layer the capability of detecting IoT threats [27]. To improve cyber threat detection in Internet of Things environments, the latest research has focused on developing Deep Learning-based intrusion detection techniques at the field’s cutting edge [28]. Cybersecurity is the space of deep learning, and defending against cyberattacks is no longer uniform. They both work together to improve and harden systems against whatever kind of evolving cyber threats are encountered. Deep learning and cybersecurity based on artificial intelligence and machine learning have led us to the modern battleship filled with high-tech and well-organized defence systems against various threats. Security specialists can prevent cyber threats, protect sensitive data, and ensure the digital infrastructure is secure using deep learning models such as DCNN, RNN, and SAE [29].

The urgency of solving the problem of dealing with the constantly growing threat of DDoS attacks in network security made me do this research. The attacks have reached the level of intensity and spread that has caused disruption to vital network services and seriously harmed an organization’s finances and brand image. The increasing ferocity of these assaults highlights the fact that better detection systems and their resolution methods are becoming a high priority to fight back effectively [30]. SDN environments being prone to DDoS attacks is a major concern as they have flexible configurations and central control, but also new vulnerabilities are another problem. This means the SDN networks require equally sophisticated DDoS detection tools to detect and filter out such attacks in the future—particularly tools designed for SDN [31]. AI-based methods, such as ML and DL, can deal with high volumes of data on network traffic and detecting patterns that are different from the rest. They have proved effective in devising ways to boost the overall security of networks. The models of SDN environments’ dynamics must be meticulously optimized, modified and changed to reach their full potential. This optimization is very important as the accurate detection of DDoS needs fine-tuning of the model to extract features, adjustment of hyperparameters, and optimization of the network architecture.

**Fig 1:**
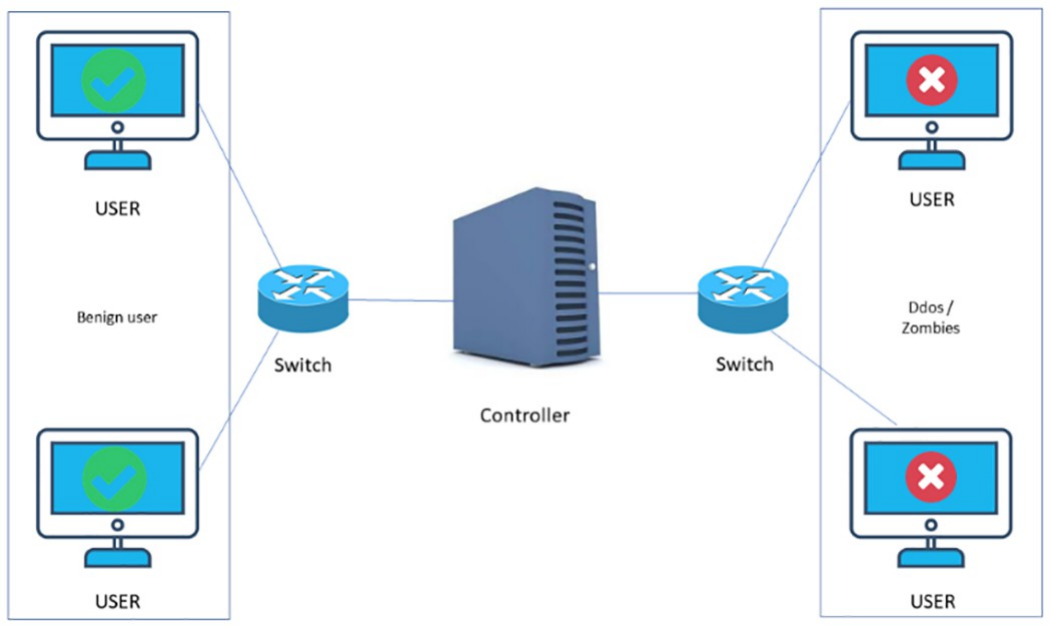
Attacks by DDoS on the SDN controller.

The customization and optimization down to the smallest detail are very crucial for increasing the effectiveness and the precision of DDoS detection in SDN environments, where the system behaviour is very different from the conventional networking environments. However, since small DDOS and SDN networks usually follow different features (for example, different structures and centralized control, detection systems must be customized and tuned for the given conditions to prevent attacks in both networks as shown in Fig 1. On the other hand, network administrators must be adaptable to adapt to situations that occur spontaneously. Also, assessing the reasons behind the detection is fundamental to improving the network management [32].

These systems should be able to quickly cope with unexpected network configuration modifications and attack methods. In particular, an SDN environment should be built specially for DDoS detection testing to evaluate the solution’s effectiveness more precisely. Such testing provides substantive data as to the model’s competence in recognizing bona fide traffic from DDoS attacks and, thereupon, defines the level of efficacy [33]. These powerful motivations are the main drive behind this study, which aims to create an ’Optimizable MLP-CNN Model’ specifically tailored to improve DDoS attack detection in SDN environments. This research proposes a stable, highly driven and transparent method that not only reinforces but also makes attacks using DDoS less probable by combining genres of machine learning and deep learning into the process with well-designed optimization.

With the mixture of DL techniques, it has become easier for the ML to pick up DDoS attacks. Convolutional neural network (CNN) and recurrent neural network (RNN) are two kinds of hybrid deep learning (DL) models that integrate their strengths to decipher varied relationships and patterns embedded in the traffic data, thereby making the dataset more accurate and reliable by increasing the chances of getting correct results. As part of the ongoing fight against DDoS attacks, machine learning shows its strengths in the way it can adapt, learn, and improve over time. Through the implementation of tools such as feature selection, optimization, and hybrid DDL approaches, ML-based DDoS detection technologies can reach higher levels of precision and effectiveness to benefit electronic networks from cyberattacks [34].

The main outcomes of the transition to SDN are numerous, though they are concentrated at the centre of the network; of distinct concern is the exposure to DDoS attacks. A DDoS attack aims at overloading a system or a network through an excessive flow of traffic originating from different sources. In the case of SDN, this centralization introduces several unique risks that threat agents can harness. At the same time, SDN has some significant weaknesses, of which the most obvious is the Single Point of Failure represented by the SDN controller. Being the core decision maker in the whole network the controller turns into an irresistible object of attack. However, if the attacker can penetrate a particular segment or overload the controller, then he can threaten the entire network. This is because all the network devices want the controller to provide a channel through which it directs the flow of traffic. An attack on the controller may therefore result in configured outages or misconfigurations across the network hence a lot of disruption. Another critical threat is Control Plane Saturation. In SDN when a device in the network encounters a packet that it does not understand, instead of forwarding or dropping it sends a packet-in message to the controller [35]. In this mechanism, a DDoS attacker is in a position to send large numbers or unpredictable packets into the network causing the network devices to forward enormous packet-in messages to the controller. This can easily drain the entire controller capabilities to the extent of failing to reserve capacity for genuine requests and additional consequential centralized network failures. This kind of attack is more risky because it hits the control plane directly while this is important for the healthy work of different networks. Two more vulnerabilities are unique to SDN, with Flow Table Overflow as one of them. SDN switches are characterized by flow tables that contain directions (flow rules) on how to process incoming and outgoing traffic. However, these tables have a very small capacity. The chief challenge to OpenFlow with regards to resilience against attacks is that the attackers can merely flood the flow table by creating a very numerous series of distinct flow networks. New flows may be dropped even legitimate ones since the flow table may start filling up again after it is filled. These are bad for the network and may eventually affect the services being offered by the legitimate users of the network.

However, these deficiencies and risks have been identified to affect the security and reliability of SDN networks, there are several design changes and solutions, which have been developed. These solutions intend to offload control processing, perform traffic filtering at the edge of the network, dynamically control flow rules, and integrate machine learning to detect threats in real-time. There has been proposed a single solution called Distributed Controller Architecture. It is also different from dedicating a single, centralized controller to the task, because it spreads the control plane out over multiple controllers. This distribution assists in fractionalizing the control load and eliminates the possibility of a single-point failure. If one controller is corrupted, or if it becomes overloaded, other controllers can take over its load, so that network operations are not disrupted. The benefit of this strategy is that it greatly enhances the robustness of the network against DDoS attacks and other threats aimed at the control plane. On this front, the next important solution is called Edge-based Filtering. Through advanced filtering mechanisms applied at the input of the SDN network, many of these attacks can be stopped before the stream gets through to the controller. This approach also ameliorates the potential issue of control-plane overload by reducing the amount of malicious traffic that is required to be processed by the controller. Some of the edge-based filtration strategies include rate limiting, anomaly detectors, and filters based on the signature to prevent attacks as they progress and reach the centre of the network.

The Dynamic Flow Rule Generation is an innovative system that deals with the flow tables of the SDN switch. Unlike most solutions which try to set up flow rules manually, this solution employs both the skill and the knowledge to automatically create flow rules and adjust them depending on the prevailing network conditions and the traffic prevailing at that given time. This assists in minimizing flow table overload by guaranteeing that only useful rules are stored in switches at any one time. It means it becomes easier to delete rules that are no longer in use or are outdated so that fresh ones can be added. This enhances the security of networks while at the same time increasing the performance and efficiency of networks. Last but not least is to incorporating Machine Learning at the Data Plane level is a revolutionary way to boost SDN security. The network devices (the data plane) are then able to use machine learning algorithms to identify aggression or threats that may be potentially detrimental to the system and does not always have to send the matter to the central controller for decision-making. This can help slice down the response time for identifying a DDoS attack or even any other security threat. Also, it makes it possible to provide localized countermeasures at the spot, which will also improve the performance and security of the network even more. The ML models can automatically learn from the various traffic patterns on the network they are monitoring, and produce better results with time when it comes to detecting between normal and malicious traffic. Thus, the article revealed that despite the numerous benefits of Software-Defined Networking as for network flexibility and manageability, its centralized structure does pose particular threats, particularly concerning DDoS. However, the above-mentioned vulnerabilities could be more or less reduced by adopting the following tenets: Distributed control architectures Edge-based filtering Dynamic flow management Use of machine learning on threat detections. Some of these improvements do not only increase the security aspect of SDN networks, but also provides for its better overall performance, capacity, and durability.

This paper proposes the CNN-MLP fully equipped optimizer, which has a DDoS attack finder using the strongest metadata in the dynamic bit selection and the DL data mining to get the right solution from all data sets. Even though a crossover neural network consisting of both the Multilayer Perceptron (MLP) and Convolutional Neural Systems (CNNs) captures the mix arrangement activity elements and conditions very well, the SHAP-feature selection strategy identifies the important features [36]. The model’s integrity can be best secured by ADAM optimizer and parameter tuning [37]. The SDN proves the model validity while executing CICDDoS2018 datasets, notably in SDN protocols [38]. Solutions for SDN security enhancement against DDoS threats are being promoted, and at the same time, CNN-MLP is equipped with an Optimizer, the optimization with Bayesian and ADAM optimizer is being detailed, and the evaluation using public datasets is up to the mark [39]. After that, the writing appears in the survey, considers basic ideas, gives a presentation, points out, and ends with offered experiences to assist DDoS detection in SDN environments [40]. This covers the intensified data mining of related literature, focusing on the reaching stage of the machine learning approach for DDoS attack detection. This reveals the evolution of DDoS attacks and how machine learning could counter them. In the following, the core ideas and the backdrop of the topic are demonstrated. The pace acknowledges the complexity of various machine learning techniques, SDN architecture, and DDoS attacks. The structure of the optical diagnostic architecture of the CNN-MLP model composed of the feature selection and combination is presented here. The neural network design, which is a combination of MLP and CNNs, is highly valued for its ability to capture complicated network traffic patterns. At the same time, the SHAP feature selection method is highly appreciated for its performance in identifying the most important attributes for DDoS detection. Through a detached study of the Optimizer-laden CNN-MLP algorithm and a comparison with recent approaches, the algorithm’s accuracy is then extensively analyzed. To tell a complete story about efficiency, several evaluation metrics, namely, precision, accuracy, and recall, are taken into consideration. Tests performed on the InSDN and CICDDoS2019 datasets show that the model’s accuracy and robustness in detecting DDoS attacks in the SDN environment is very high.

In the last part of this paper, we spotlight the Optimizer-equipped CNN-MLP model contribution towards Sand-storming the SDN Security against DDoS attacks, and he also indicated the directions for future research. Besides defining the very case, it as well stresses the context of the pre-emptive measures against cyber-attacks in the SDNET and through the cooperation with academy, industry and regulating agencies it’ ultimately promotes the continuing efforts to address the matter. Here, we explained why the Optimizer-equipped CNN-MLP display has certain drawbacks and is made by the proposal for future improvements. Productivity and accuracy of discoveries can be enhanced using different DL structures, fine-tuning the hyperparameter tuning forms, and improving highlight selection techniques.

### 1.1 Related work

Besides, the cloud-based architecture associated with SDN makes it vulnerable for cyberattacks due to rapid development in wireless communication, such as IoT [41]. DDoS attacks are the most common and dangerous type of attack in this area. In this regard, deep learning (DL) techniques are growing in popularity among researchers who use machine learning (ML) algorithms for anomaly detection to solve this challenge, generally due to the good results they have demonstrated. The DDoS detection process has shown nothing but effectiveness through the striking feature of the centralized SDN control plane [42].

Several researchers have proposed different ML-based methods to detect distributed denial of service in SDN environments. Not only, in a multi SDN controller environment, just a Naïve Bayes classifier but it also represented DDoS at-tacks with human accuracy [43]. Just like that, Tang et al. [44] put DL techniques in a spot-light, using GRU for network anomaly detection utilizing NSL-KDD and CICIDS2017 datasets for up to 99% precision gain [45]. Nevertheless, the fact that the NSL-KDD dataset is packet-based has made its practicality for use in SDN environments questionable. However, similar to Dey et al. used the DL techniques, the effectiveness of flow-based detection for the high accuracy of the DL techniques was very poor [45]. The current limitation of individual models can be overcome by ensembled versions, which, in turn, can help reduce biases and deviations while increasing accuracy. Haider et al. developed several ensemble DL models, such as RNN, CNN, and LSTM, and they achieved the accuracy of above 99% attacks for detection [46]. Ensemble CNN models with higher computational load, however, were found extremely precise in discovering DDoS attacks as many investigations confirmed them [43].

Moreover, there is a promising way of designing a system using a hybrid method, one which essentially combines deep learning with a machine learning classifier. Sharma and other researchers’ real-time intrusion detection system, which combined several layers and an ensemble method, could detect multiclass attacks [47]. Mhamdi et al. [48] dealt with the imbalanced training data by using the improved conditional variational autoencoder to solve the problem. However, Reference [48] used autoencoders along with the One-Class SVM to detect anomalies. For example, other solving methods involve this fast KNN binary classifier to deliver an effective voting scheme that can quickly and reliably identify attack traffic [49]. Another possibility entails the integration of history-based IP filtering and self-organizing maps, which, in turn, can emphasize the ability of the SVM method to detect attacks accurately [50]. In the process of limiting communication capacity in SDN controllers, feature selection techniques have also been proposed, which resulted in improved accuracy and reduced training time [51].

In addition, for SDN environments, the MLP, as well as an optimizer, adds to the capabilities of detection mechanisms to resist the Distributed denial of services. The basic neural network architecture called MLP forms the backbone of the system used for analyzing complex network traffic data and detecting DDoS attacks that can be made by spotting important patterns in the data. The MLP model with an adaptive moment estimation optimizer can learn to classify the normal flow of legitimate traffic from DDoS attacks. The most realistic MLP for DDoS detection is an adaptable and adjustable device that can suit various network conditions and develop according to the changes in threat panorama. The MLP is the key component of the SDN network’s defense against possible DDoS attacks because it can learn from the large dataset and detect the smallest abnormalities in the network traffic.

Placed with restless reiterations regarding loss reduction and improved accuracy, the optimizer empowers the MLP model and elevates the general efficiency of the process of DDoS detection. The optimizer and MLP work in a symbiotic way that helps to develop a DDoS detection system that is more reliable and effective by using each other’s advantages. Adjusting the parameters of the models and enhancing the convergence rate is the optimizer’s primary function, which complements MLP’s inherent ability to find complex patterns the data holds. Highlighting the complementary nature of the described approach gives network administrators extra important and dependable threat intelligence, including fewer false positives and false negatives and improved DDoS detection accuracy. The most efficient way to improve DDoS detection mechanisms in the SDN environments is using MLP and an optimizer. MLP is potent in pattern recognition and optimization and is able to adjust the model parameters to make organizations more prepared for DDoS attacks.

In [52], the more gadgets are connected to the network, the higher the potential of DDoS attacks, which is why traditional models of detection are ineffective, especially given the highly fluid nature of IoT networks, and the requirement for immediate response. Based on the previous studies, this study concludes with the construction of a decision tree-based decision model especially designed for DDoS attack detection with the hope of improving detection accuracy for intelligent information systems. The paper explains step by step the experimental framework used in the research, including data pre-processing and applying different libraries of machine learning, including Sklearn, Keras and TensorFlow. In particular, the accuracy of the model reached 99% when tested for predicting DDoS attacks, which might mean its applicability for practicing. The authors of the paper sum up their work with informative remarks concerning the application of machine learning algorithms in DDoS detection and highlight the urgency of a deeper investigation of security issues in IoT settings.

The study [53] is to identify and choose a suitable dataset that captures temporal and spatial aspects of DDoS attacks and traffic data for developing a range of machine learning models for categorizing the prevalent forms of DDoS attacks. The study used the following approach: Data pre-processing involves data cleaning and data transformation, and then the authors proceed to adopt feature selection methods. First, the dataset had 87 features, out of which using an Extra Tree Classifier model to check the important features needed for classifying the data. The analysis considers six machine learning subject areas namely, Random Forest, Support Vector Machine, Naive Bayes, Decision Tree, XGBoost, and AdaBoost. Out of these, AdaBoost provided a result of 99.87% accuracy at a computation time of 27.4 sec. The study reveals AdaBoost and random forest classifiers yielded the best results with a RoC of 1.00 for all the identified cyber threats. The authors concluded that the proposed framework can identify various categories of DDoS threats and regard the significance of developing and flexible solutions against the future DDoS threat. In conclusion, this research provides useful knowledge for future works regarding the used of feature selection and multi-classifiers for boosting the detection and prevention of DDoS attacks, which is a crucial problem in the modern field of network security.

## 2. Methodology

The principal merit of the proposed project is to make Software-Defined Networking (SDN) environments more secure by enhancing their ability to detect and counter Distributed Denial of Service (DDoS) attacks. DDoS attacks are a common threat to modern network infrastructures as they can overwhelm servers or networks with malicious traffic, making them unavailable to authorized users. Since SDN systems are continually changing and complicated, regular security measures might not be adequate to deal with these attacks. Thus, more enhanced and accurate systems that can promptly identify and counteract DDoS attacks are required to ensure the uninterrupted running of the SDN infrastructure.

A multimodal approach and new methods from machine learning and neural networks solve the issue of this proposal. One of the important aspects of the plan is the feature selection technique called SHAP (shapely Additive explanations) feature selection, which is the one that chooses the most significant features for DDoS detection. Through this process, the interpretability of the model is improved and the threat detection accuracy is increased by focusing on the most important components of network traffic. To complexly process the patterns and spatial linkages of the network data, the model combines CNN and MLP architectures. This combination boosts the model’s feasibility of finding small DDoS-related changes. Besides, combining Bayesian optimization with the ADAM optimizer makes the hyperparameter tuning process more efficient thus making it easier to find the best configurations that improve performance. This paper aims to increase the network system safety against dynamic attacks and enhance DDoS detection capabilities in SDN settings by proposing a model incorporating these cutting-edge technologies.

### 2.1 Feature selection

We used two separate datasets, CICDDoS-2019 [54] and InSDN [55], for our research. It is as, [54] says CICDDoS-2019 encompasses many DDoS attack traces, mostly TCP/UDP-based rules at the application layer utilized for DDoS attacks. DDoS attacks are divided into two primary categories in this dataset: The incidents that tend to occur in the forms of attack are exploitation attacks and reflections attacks. The first one hides the real IP address of the attacker by using a reflector server to redirect the malicious traffic to the intended victim. In contrast, the second one is unnecessary to use the reflector server and thus goes directly to the victim. The CICDDoS-2019 dataset, consisting of 88 different features, contains numerous facets. Nonetheless, the SDN infrastructures are the heart of the InSDN dataset, which the authors describe in detail in [55]. The smartly constructed dataset was selected by the use of four virtual machines which were put in place. The attacks in the dataset were done by external and internal sources from within the SDN network. The traffic monitoring system needs a complete view of the regular traffic patterns that cover the applications of many services. The number of 361,317, 292,893 incidents of attack traffic plus 68,424 harmless or ordinary traffic incidents shows how many of those 292,893 attack traffic incidents and the 68,424 harmless or ordinary traffic incidents there are. We can conduct our research activities using the eighty-four main features in the dataset.

### 2.2 Feature selection

Shap-feature selection, which is the main component of SHAP, is the Shapley value which is the high-end method of the feature’s importance in the predictive model [56]. It gives each component a digital number that shows how much of a job it does in the model’s prediction, based on the ideas of cooperative game theory. This score is created by cautiously observing every possible feature combination and then checking its impact on the model’s output [28]. A trait with a large SHAP value significantly impacts the model’s output. Data scientists can easily determine which features are the most significant for people’s comprehension, and the models are accurate using the SHAP values, which would, in turn, help the finder identify the most important features [40]. To begin with, we see cooperative games in which the players cooperate to achieve the objectives and thus, to find the Shapley value of a certain feature. Further, every part in this situation plays the role of a player and thus affects the model’s predictions. The Shapley value analyses all the possible combinations of the features that consist of or are not considered, and thus, it assigns the contribution of each feature. The Shapley value is computed by the difference between the value of the groups consisting of features other than the feature in question and the total value of the groups that do not include the feature. Besides, the total number of players (features) is divided by that computation to get the estimate of the contribution of each feature to the coalition as a whole. This is how the formula is expressed: This is the formula that is stated in this way:

$$\text{psi}\left(\text{w}\right)=1/\text{s}\;\;\sum \text{T}\subseteq M\backslash \text{i}\{\text{n}-1\left|\text{T}\right|]\;(\text{w}\left(\text{T}\cup \text{i}\right)-\text{w}\left(\text{T}\right)$$ (1)

Where T: Cumbers is the abbreviation for the number of participants in a coalition or the size of the coalition.

M: It is the total sum of people in the game.

The value of the coalition T is represented by w(T). This number is the total bonus or benefit produced by the members of coalition T by working together.

i: The game characterizes a certain player.

The summation in Eq (1) is the symbol for the sum of all possible coalitions that do not involve a particular player, player *i*. The worthiness of each coalition T is shown by the worth w(T).

### 2.3 Multi-layer Perceptron (MLP)

An MLP is a specific category of ANN that can be used in various machine-learning processes. It’s been frequently employed in cybersecurity, recognizing distributed denial-of-service (DDoS) attacks in software-defined networks (SDNs). An MLP comprises more than one layer of nodes or neurons, each of which begins to process the input data and then passes it on to another layer for processing. This architectural design allows MLPs to learn structural and dependency information about the data; therefore, they are useful in discovering patterns, such as recognizing DDoS attacks [29]. However, the static pattern in session data for detecting DDoS in SDNs is another significant advantage of employing MLPs [43]. This can be done in real-time and enables the model to improve its learning capability to detect emerging patterns in network traffic, making it possible to detect new DDoS attacks. Moreover, it has also been established that MLPs achieve a higher level of detection accuracy than traditional methods. They do this by analysing historical traffic data and identifying common traffic patterns within a network. Then, they can detect ‘unfamiliar’ traffic patterns or traffic deemed malicious with reasonable accuracy [57].

MLPs also proved to be resource-efficient for the detection of DDoS, thus further promoting their usage. DPI techniques, in general, can be computationally intensive and demanding on memory, while MLPs’ impact is significantly smaller. For this reason, they offer the possibility of becoming a practical solution for organizations that employ cheap means of implementing DDoS defence. However, it is important to note that for the same set of transforms, MLPs can be coupled with feature selection algorithms as well to achieve the desired levels of efficiency [58]. These techniques contribute to the narrowing down of relevant features within a set of given datasets, which in turn contributes to the high-level reduction of dimensionality and subsequently makes the DDoS detection model even more accurate. This paper points out many benefits linked with incorporating MLPs in practice regarding implementation in SDN architectures. Their characteristics such as scalability, flexibility can easily extend working into the current network infrastructure, allowing the process of real-time DDoS identification and removal [59].

**Fig 2:**
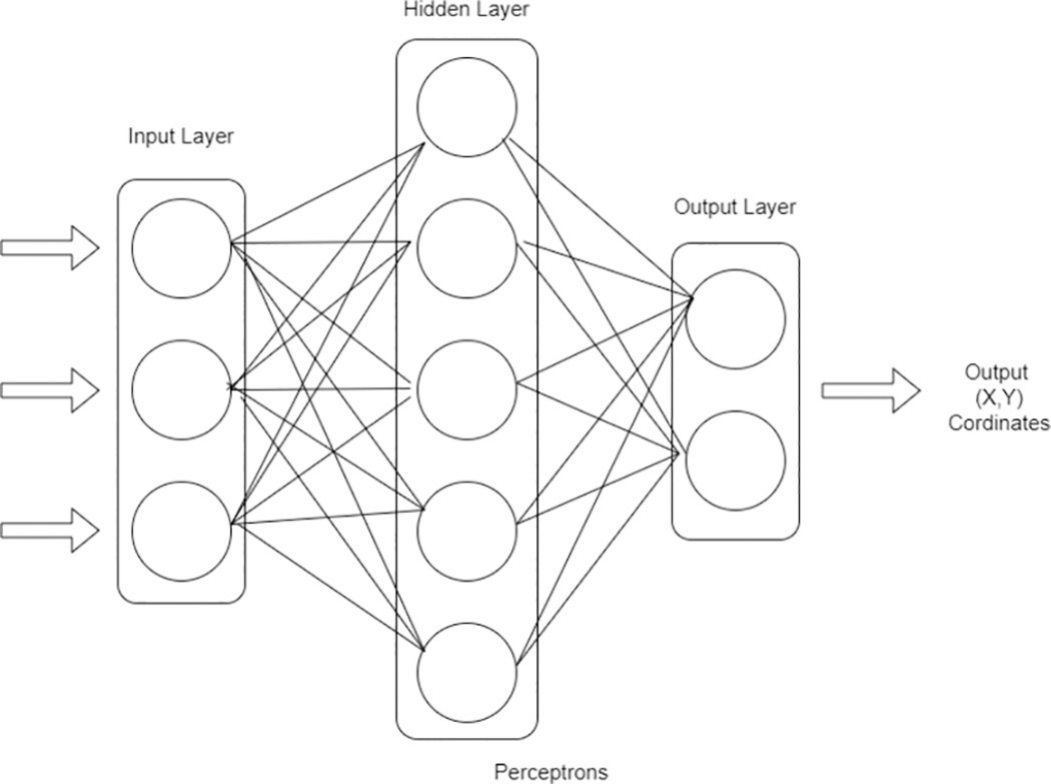
MLP framework.

The Multilayer Perceptron (MLP) depicted in Fig 2 is the basic part of the deep learning models that assist in many machine learning tasks, including feature extraction, regression, and classification. MLPs are a special form of a feedforward neural network that lets the data to travel from the input layer to the output layer in a single direction only. We ook into the details, functions, and the way MLPs are structured in more depth [60, 61].

**Fig 3:**
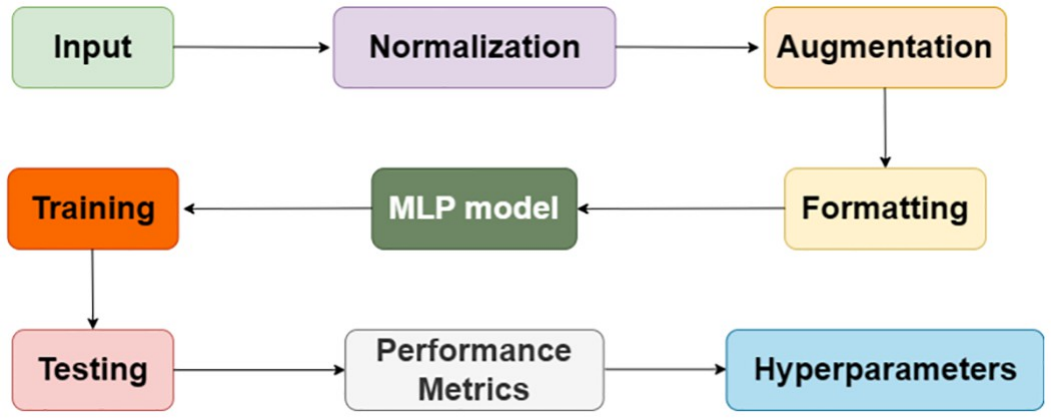
MLP framework.

Let us examine each step of the MLP design of stage 2, which is given in Fig 3. Before building an efficient MLP model, the first step is pre-processing the data. This encompasses the whole spectrum of activities, from scaling the data so that they are comparable, formatting the dataset to make sure it meets the requirements of the model, and augmenting the data (usually in the picture data) to enhance its diversity. We first have to state the architecture of the MLP model, and then we can start designing it. Three layers, namely, the input, hidden, and output layers, have been combined to make up the model’s three layers in this case. Every layer assembles the data and produces the predictions in a specific manner. The layout of the input layer is the consequence of the size of the dataset’s features, as shown in Fig 2. Because each input layer neuron is a feature, the model extracts the required information from the dataset. In the deep layers, where most of the processing happens, many neurons use the activation functions to change the data that comes. The ReLU function in this suggested architecture is used for activating 128 neurons in the first hidden layer and 64 neurons in the second hidden layer, as shown in Fig 2, using the same technique. ReLU was picked because it gives nonlinear behavior, which the model needs to catch the complicated patterns in the data.

The structure of the output layer, as shown in Fig 2, is at the mercy of the job in which it is used. Usually, one neuron is placed in the output layer of each class in a multi-class classification, and the softmax activation function generally obtains class probabilities. A neural network with a sigmoid function activation can create the probability of one class in the binary classification. The second step in the model building process is to assemble the model architecture that has been previously described. This signifies that the evaluation metric, loss function and the optimizer are to be detailed. The optimizer modifies the model weights to the minimum loss so that the model can be tested in the training process. The assessment metrics are the hints of the model’s workability that are revealed by the validation dataset [61]. The final Eq (2) of MLP is given below.

$$a_{j}^{p}\;=f\left(\underset{i}{\sum }m_{ij}^{p}\;b_{i}^{\left(p-1\right)}+c_{j}^{p}\right)$$ (2)

Where $a_{j}^{p}\;$ is the activation of neurons j in layer p, m is the weight, b is the output of the neuron and c is the bias and f is the activation function. Once the model is built, it is most commonly trained on the training dataset for several epochs. During the training phase, the model learns how to predict by adjusting its weights depending on the data given and the loss function. The practical training is the next step after introducing the model in which the model’s performance is evaluated using a different dataset. The performance indicators, which are accuracy, precision, recall, and F1-score, are used to check the way the model fits the new data. Not only should some models be made or new data have to be collected, but hyperparameters like batch size and learning rate may also be changed. Therefore, the parameters are be modified based on the model’s efficiency in the validation dataset. The model can be used in real-life situations once tested and proven to perform well. Nonetheless, the models’ effectiveness get worse over time. Hence, regular reviews and updates are needed because of the data distribution getting shifted or any other reason that might cause the models’ performance to be worse.

**Algorithm 1**:

The MLP Architecture This paper proposes an MLP architecture, which is a Multi-Layer Perception that is be used in analyzing the data.

Require: The training data or data set used in building the speech recognition model is known as Dtrain, and the testing data or data set used in evaluating it is known as Dtest.

Require: parameters of the solely constructed MLP structure

 1. Prepare the data sets further: normalize the data, extend the data set with augmented data, and format the sets.

 2. Construct a multiple linear regression model:

 3. There are 3 layers.

 4. Units for each layer.

 5. The first neurons in the network are the inputs neurons that directly correspond to the features of your data.

 6. Hidden layer 1: The first hidden layer of the ReLU activation function has 128 units.

 7. Following the first hidden layer, there is the second hidden layer as follows.

 8. Output classes are the next relevant layers, which should employ an appropriate output activation function such as softmax.

 9. Depending on the type of problem–binary or multi-class, respectively–some functions like softmax, sigmoid.

 10. Before constructing the MLP model, it is crucial to define the type of loss, the required optimizer, and which measures are used for evaluation.

 11. The idea of the next step is to adjust the MLP model to Dtrain, or in other words, to learn MLP model supervised on Dtrain during the specified number of iterations.

 12. The distest is a perfect tool to compare the model’s performance and test it there.

 13. In case it is necessary, one can increase or decrease the values of the hyperparameters for a certain model to meet the desired learning objectives.

 14. You have the option of submitting the just-created model, for example.

 15. They also need to monitor and update it as it is a continuous process QE: It can also be constant their watching and altering.

### 2.4 Convolutional neural network (CNN)

CNNs are one of the most widely researched and used deep learning approaches suitable for image, video, and text analysis and various industry tasks, such as cyber security. Primarily, particularly in Software Defined Networking (SDN), CNN is useful in identifying Distributed Denial of Service (DDoS). If one aims to understand how CNNs work to address the threat of DDoS attacks, then it is essential to consider the two and explain the general principles of their functioning [62].

CNNs consist of four essential elements: Some of the main components of a CNN include convolution layers, Pooling layers, activation functions, and fully connected layers. Convolution layers are used for weights data that contain some spatial structures like images or flow data like packets in a network [62]. Pooling layers then reduce the dimensional extent of the analyzed data while preserving essential details and, as a result, improving the model’s performance. Activation functions limit the inputs given to the neurons, enabling the model to learn complex relationships between the input characteristics. Last but not least, the fully connected layers separate generalized features based on pre-defined classes to differentiate ordinary traffic from DDoS attacks [63].

**Fig 4:**
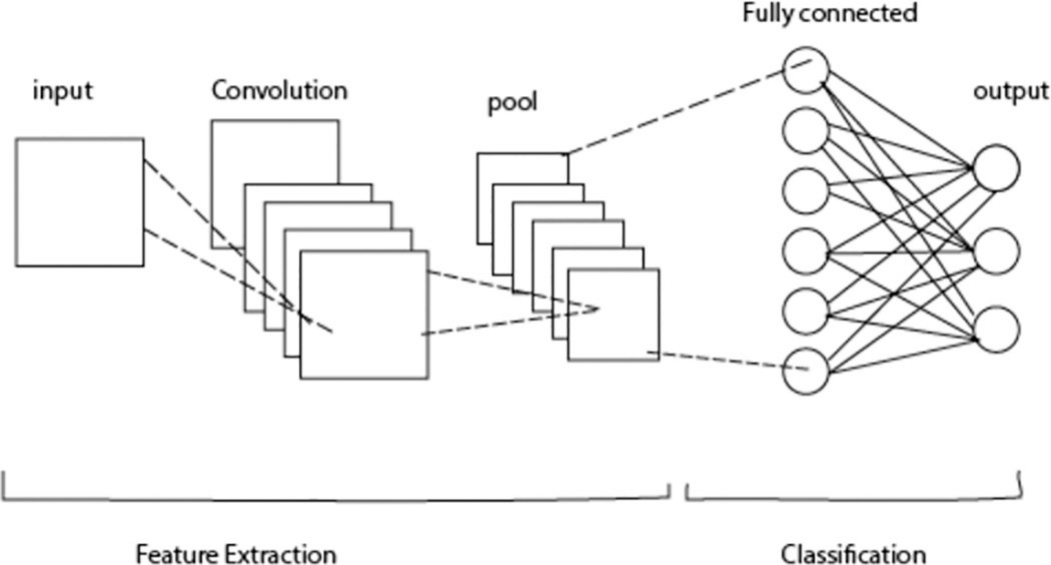
CNN framework.

Using CNNs to detect DDoS is beneficial for many reasons because of the capacity of CNNs for feature extraction, patterns identification and classification. First, the feed-forward CNNs can identify expected and relevant features from raw image inputs, which is generally complex and high-dimensional in network traffic data. The convolutional and pooling layers in CNNs as shown in Fig 4 help the networks detect small changes, which suggests that there might be DDoS attacks [33]. Second, the article shows that CNNs have outstanding performance in analyzing various patterns within the feed, which in the case of DDoS attacks means better recognition of attack signatures that could range from sudden spike in traffic flow or abnormal distribution of packets. Finally, utilizing fully connected layers, CNNs classify the identified patterns as either belonging to normal traffic or the malicious one characteristic of DDoS [64].

Applying CNNs in DDoS detection has several benefits over traditional approaches, Sanchez et al., [65]. For example, reinvented CNNs have higher accuracy rates than other machine learning algorithms, often surpassing those of classical models. Also, CNNs have relatively high robustness to noise contaminations and reasonably optimal performance even under varying network conditions, making the models efficient for real-world use. The fourth and final worthy-of-note feature is the capability of CNNs to scale to accommodate expansive data sets produced by large-scale SDNs.

**Fig 5:**
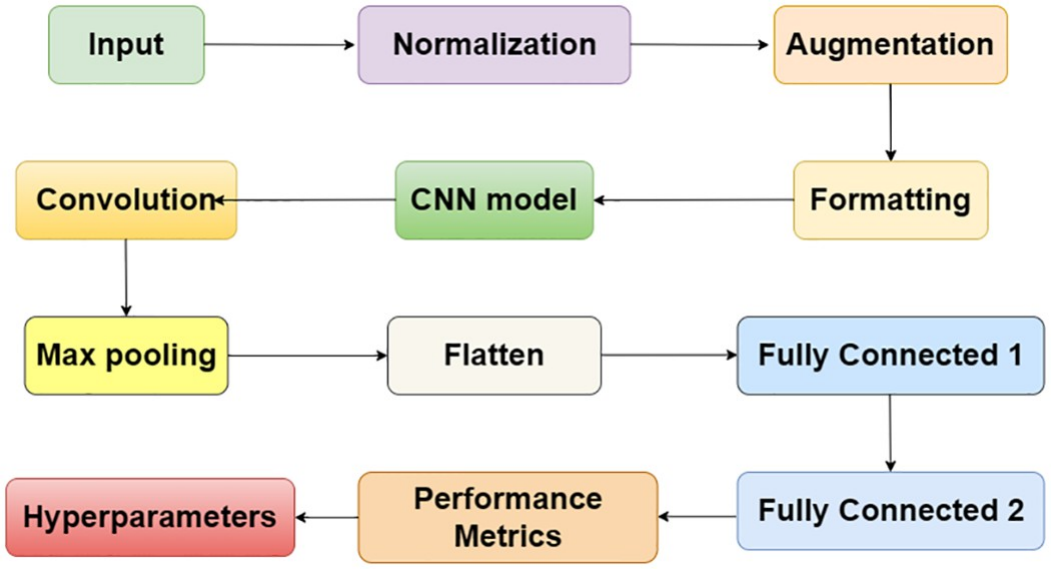
CNN frameworks.

Through the pre-processing of the data, which consists of formatting, augmentation, and normalization, the proposal of the CNN architecture, the dataset is ready for the training. The four types of picture data processing, normalization, formatting, and augmentation are used for the scaling of data to a similar range, the formatting of the data for its appropriate display, and the augmentation of the dataset to increase its variety. After the CNN model is set up, the CNN model is developed. They include convolutional, max-pooling, flattened, and fully linked layers, as shown in Fig 5.

The first layer, shown in Fig 4 of the convolution, comprises thirty-two three-by-three-dimensional filters. The same padding keeps the space dimensions, and the ReLU activation makes the nonlinearity. The feature map reduction is achieved by max-pooling a two by two pool size. The second convolutional layer contains 64 filters, the same as the first one. A second time, max-pooling and the same padding are also present after the ReLU activation. The flattened layer, which is the layer adjacent to the convolutional network as shown in Fig 4, is the one that takes the 2D feature maps and transforms them into a 1D vector, thus allowing the fully connected layers to deal with them.

The output layer, as shown in Fig 4 consists of the same number of units as the classes in the dataset 128 for the second fully connected layer) when ReLU activation is applied. Even though the sigmoid activating function is used to classify the binary data, the softmax activating function is applied in the multi-class classification. Finally, the CNN model is completed, hence the optimizer, the training evaluation metrics, and the loss function. The model changes its weights to minimize the loss for all the epochs that the model goes through the training on the training dataset. The model’s performance is evaluated by using several metrics from the testing dataset that are applied after the training. Parameters can be modified more to achieve a better outcome in this study.

The model can be applied to several cases. The progress of a process has to be repeated at least once a day, and each time this happens, the data and needs are changed, and the model is modified. It should be checked every day to ensure that its long-term operation is being conserved. Therefore, in a nutshell, this method precisely describes how to build, train, test and further improve a CNN model for a certain dataset. The second point is that it is how to keep and promote the model continuously.

**Algorithm 2**

This gives the regularisation weights and the proposed CNN architecture for the simulation.

Require: The set of training examples is represented with Dtrain, while the set of testing examples is represented with Dtest

Require: environments that characterize the foundational architecture style of the Convolutional Neural Networks

 1. Clean out the data, extend it, and format the provided data to feed the classification and detection models.

 2. In total, the CNN model includes six layers, four to six of which are the convolutional layers.

 3. The first convolution layer of the extracted form: 4, with thirty-two filters.

 4. In the case of 3x3 filters and what follows, the implementation involved these filters.

 5. ReLU is the activation function

 6. Cushioning: identical

 7. Level 1 entails operating on selected elements of an image and raising some of the near values to a single, higher value without touching the other values.

 8. Availability of pool: 2, reference; 2.

 9. Convolutional layer 2:

 10. Filter types: N° Of Filters:

 11. About the filter size, I only observed in the instructions that it is written as ‘3 3’ in the puzzle.

 12. Activation function: ReLU

 13. Padding: same

 14. Then comes the second max pooling layer, whereby the features of the image are down-sampled to consist of only the essence of the image being taken.

 15. Originally, the saying ‘two by two’ pool referred to the way in which Noah loaded the animals onto the ark.

 16. Flatten layer: This compacts all the layers hence rendering it into a single long vector of features that are arrived at by squaring up all the layers that were outreached from the convolutional layer.

 17. Layer 1 fully connected in the RBM, the first layer is connected in a fully connected manner where every node in the layer is connected to each node in the next layer Although the input layer doesn’t possess the node it is also connected in the same manner

 18. The following deductions can be made with regards to quantity of units:

 19. Activation function: ReLu

 20. The layer with complete connectivity 2 is the last layer and is the model if the first layer with complete connectivity is the initial layer together with the input data.

 21. Units: class units

 22. If the classification problem is multiclass, the activation function used is softmax, and if the classification problem is binomial, the activation function used is sigmoid.

 23. Construct the CNN architecture: explication of losses, optimizers, evaluations

 24. Employ more epochs to fine-tune the CNN model on Dtrain to at least the number of expected epochs

 25. Finally, the model is tested on the Dataset and reported on performance measures such as precision, recall, F-score, and accuracy.

 26. Last of all, adjust, if needed, which are systems’ parameters or are more often known as hyperparameters.

 27. Then it can be used optionally:

### 2.5 Combined CNN and MLP

**Fig 6:**
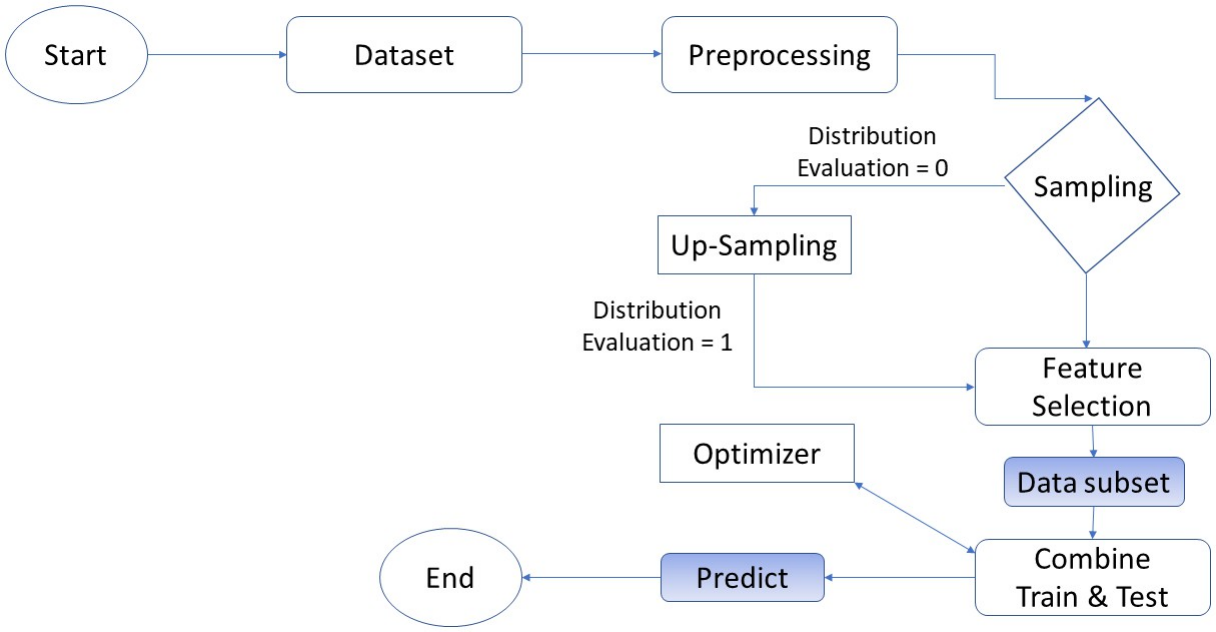
Proposed model.

The Combined CNN-MLP Detector merges the advantages of both the CNN and MLP designs, thus improving DDoS detection. The first stage in preparing the dataset for analysis is pre-processing. For this to be possible, the data must be structured, enriched, and normalized. Fig 6 shows the improvement of the model’s effectiveness through the SHAP (Shapley Additive explanations) feature selection, which allows us to find the most important characteristics connected with DDoS attacks. The data mining process starts with a dataset that forms the basis of the analytical foundation. The pre-processed procedures clean up this data; for example, the outliers are solved the missing numbers are filled up, and the format is correct for the analysis. The first phase of the assessment process studies the spread of the data to check if there is any skewness in it. This evaluation might result in the data being sampled, which would, in turn, be more efficient because it would only select the representative subset for further analysis. The disbalance of the data distribution can be solved by using up-sampling to make a balanced dataset. Once any changes are made, the data distribution is reassessed. The primary data properties that are even more relevant are selected in the next stage, feature selection, to improve the mining process. Thus, the data is used to train a model and consequently, a mathematical model for pattern recognition within the data is generated. The model’s performance is then assessed using another part of the data.

After the training, the data can be merged for more analysis or model development. After that, the optimizer changes the model to improve its performance. Sets of the data can be used for a certain purpose. Ultimately, the trained model used to predict the new data is thus the applied information from the mining process. This finally ends the meticulous evaluation of the data and conclusion, as Fig 6 demonstrates. The model aims to develop a hybrid model combining MLP and CNN architectures. This hybrid method combines MLP’s structured data handling with CNN’s picture analysis and can decipher the complex DDoS assault patterns.

The model compilation, which is the phase of creating optimizers, loss functions and evaluation metrics, follows the model development. After that, the model’s precision in detecting DDoS indications is improved by changing its parameters over some epochs and taking the training dataset as the input. The model’s performance after training is evaluated by applying the same dataset used for training. By optimizing hyperparameters, bayesian optimization enhances the model’s ability to detect and counteract DDoS attacks. After the hyperparameters of the best model have been discovered, the model is retrained using them. Thus, it could be combined with the systems for DDoS detection, which are equipped with features for checking and updating to deal with attacks that change over time successfully. The algorithm gives the highest importance to reporting and alarm mechanisms for alleged DDoS attacks so that timely responses to possible threats can be guaranteed. A complete record of the model’s design and training protocols is also kept for the reasons of the permanent record. The Combined MLP-CNN Detector that works with the adaptive features and the benefits of the MLP and CNN architectures provides a solid solution to DDoS attack detection, and at the same time, it can manage the security threats that are changing.

## 3. Performance evaluation

First, we explain the experimental setting and the evaluation criteria used to measure the model’s efficiency. After that, we test different activation functions and input weight ranges to find numerical values that work for the model in different conditions. We validate our model by contrasting its results with the state-of-the-art methods. Python was used in the experimental phase. We used Jupyter Notebook and trendy instruments like sci-kit-learn and Pandas Matplotlib. The computer environment was built so that it came with an 8 GB RAM, a 4 GB AMD RX 550 graphics card, and an Intel i7 processor. This study proves that our suggested approach is effective and stable.

The proposed hybrid model of Multilayer Perceptron (MLP) and Convolutional Neural Network (CNN) in detecting DDoS attacks in Software-defined Networking (SDN) environment s shows promising results with regard to accuracy. However, there is a set of issues with the implementation of this model in practice which should be considered. The two fundamental issues pertain to computational complexity, amount of RAM, time needed for inference, and the model’s applicability on networks with many nodes. One of the problems found with the hybrid model is that it is highly computationally intensive, especially in the training process. This requirement is a problem, especially when thinking about solutions adapted to local area networks in organizations with minimal resources. The storage of model parameters and feature maps particularly that of the CNN component poses a challenge for SDN switches or controllers that may not have adequate memory. While the model provides near-perfect accuracy, the number of operations required with the model has significantly increased inference time and could jeopardize the timely identification of attacks and subsequent response–a critical function in any security fighting force. Thus as size and load of the network increases the computation complexity of this proposed model goes on increasing which makes the model less scalable and practical in larger more complex SDN networks. These difficulties raise questions on how to achieve a better ratio between model performance and realistic advancement potentialities.

To overcome these challenges, the following strategies are proposed to enable the firm to achieve the optimum level of output for the least cost. One of them is the blocking of model parameters, for example, by pruning and quantization methods. Pruning is the process of eradicating unwanted edge or even neurons to the neural network while on the other hand quantization will downgrade the precision of numbers utilized in the model as a method of conveying the figure. Such technical procedures can reduce model size and computations to a considerable extent; however, the general accuracy may drop a little. Another proposed solution strategy is data parallelism all over the structure of SDN. It involves spreading data processing across the network and taking more computing resources nearer to the information sources. To continue, the use of specific processing circuits, including GPUs or TPUs, enables significant optimization of training cycles as well as inference time, which inherent the problems of computational intensity and response time. Moreover, with the proposed approach of adaptive model selection, its performance should be optimized based on the given network environment. This approach involves using the complex hybrid model together with simple models and where the network circumstances and perceived threats change, shifting to the use of these simple models. In this way, the system can be fast in delivering its results if a less accurate but faster model is needed while it can also use the hybrid one when it is needed. These strategies are believed to be critical for the actual deployment of the hybrid model in realistic SDN settings. They meet the major constraints of computing resources, memory requirements, time for inference, and model’s extensibility to practical use.

To determine its performance, the proposed CNN-MLP with Optimizer model was evaluated on two different datasets, CICDDoS-2019 and InSDN. The model has to find the CICDDoS-2019 dataset to get the final accuracy of 0.9995(99.95) and the InSDN dataset and the technology can show results with a phenomenal precision rate of 09998 (99.98). The research has revealed an outstanding accuracy rate. The lengths of these correctly classified instances show how well the OPTIMIZER+ CNN+ MLP model prevents DDoS attacks from a bunch of datasets. The impressive performance of the model here makes it possible for such an approach to be incorporated as the source of strong cybersecurity products used in different network environments. We have been amazed that the CNN-MLP model combined with the Optimizer is the best in our performance evaluation; for the InSDN dataset, an accuracy of 0.9998 (99.98%) has been achieved. This demonstrates how the model can access traffic information by network traffic classification. Accuracy is of great value, so we minimize the false positives. The Optimizer-equipped CNN-MLP model is the most precise in the InSDN dataset, with a precision of 99.99% testifies to the ability to reduce the rate of benign traffic being erroneously marked as DDoS attacks.

**Fig 7:**
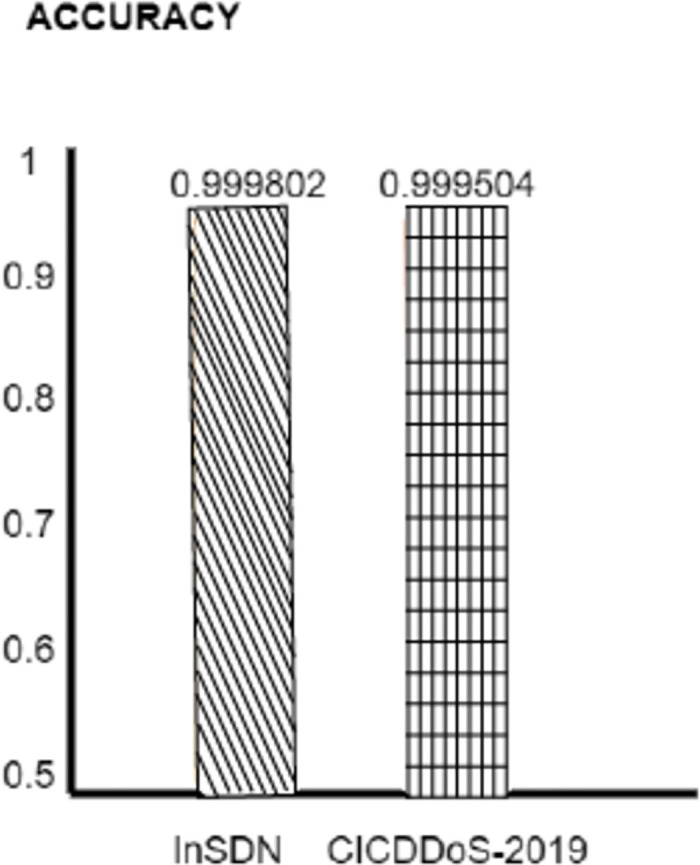
Accuracy.

**Fig 8:**
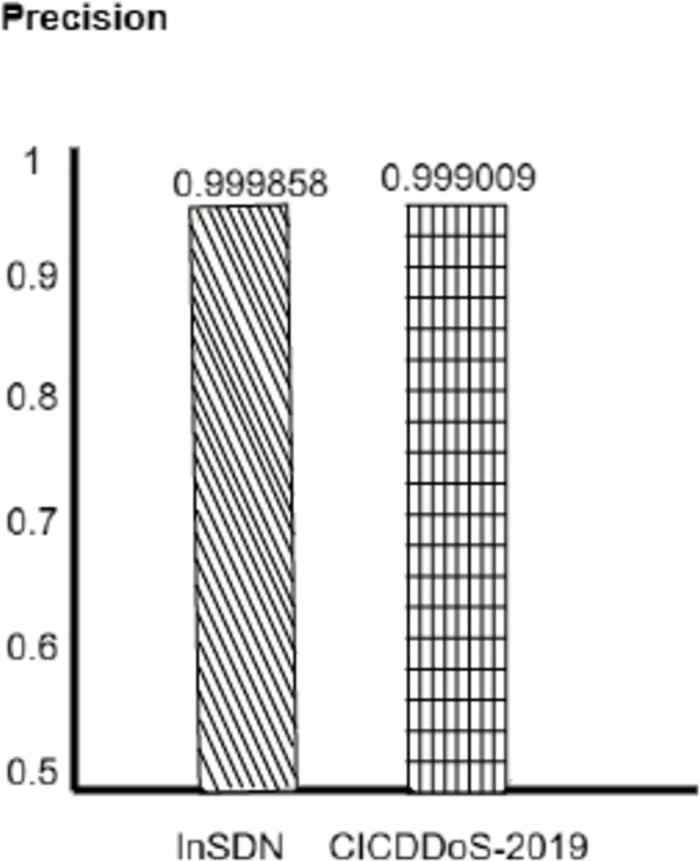
Precision.

**Fig 9:**
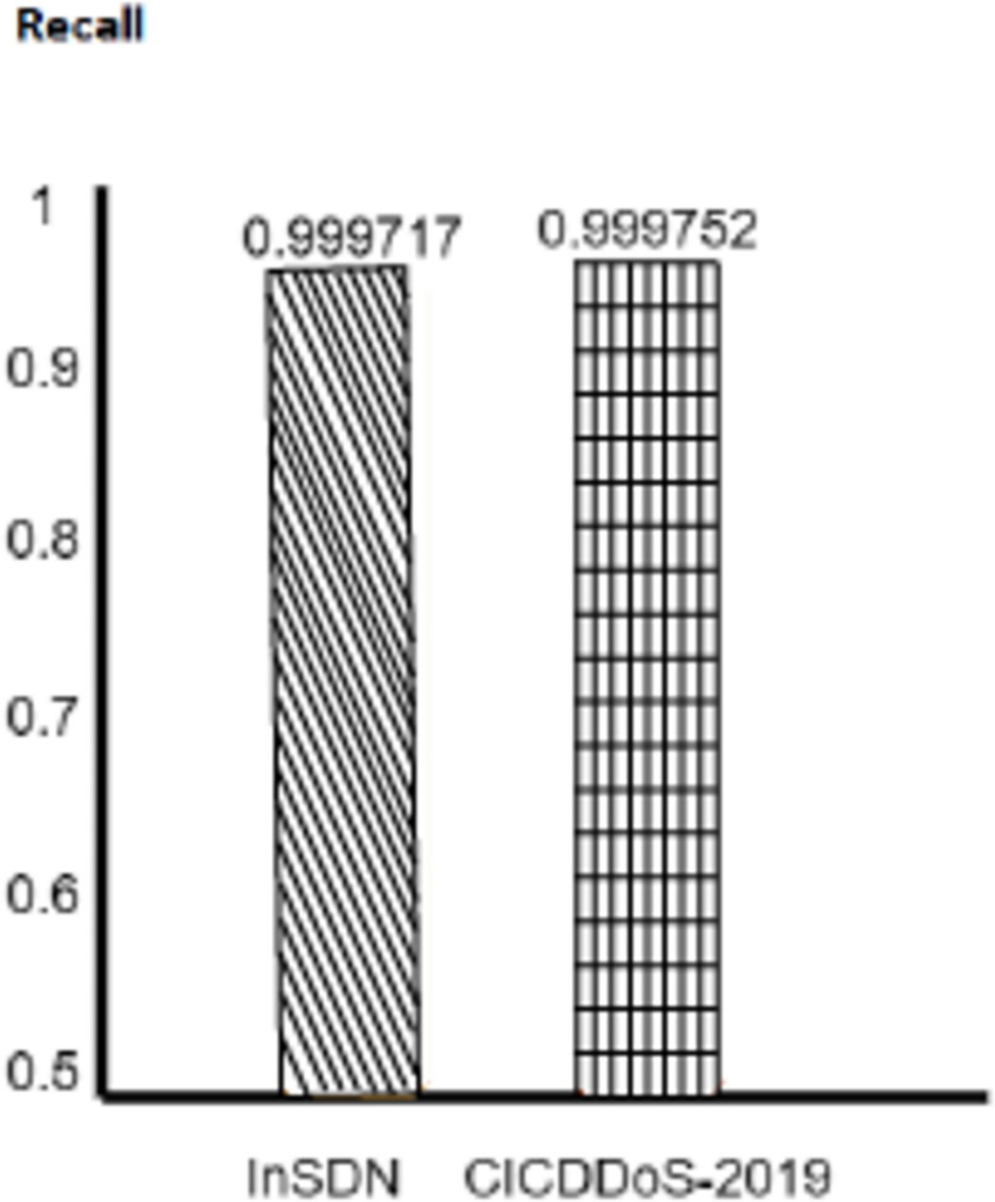
Recall.

**Fig 10:**
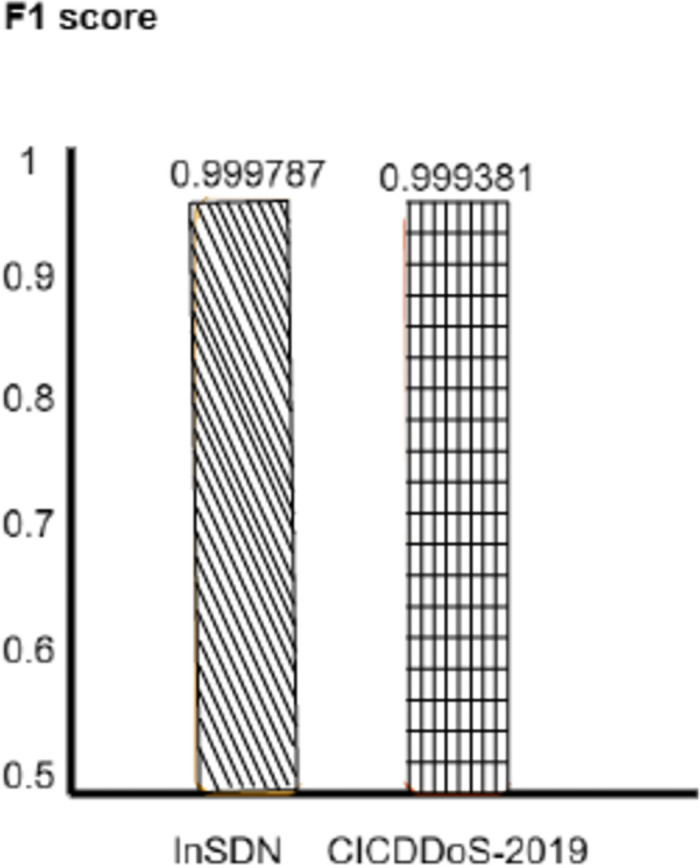
F1 score.

We evaluate how well benchmark models can get DDoS attacks in the real world, showing promising outcomes. Moreover, the CNNMLP model with the optimizer fits also excels in the recall rate and records 99%. However, InSDN has evaluation data accuracy of 97% recall. 99.98% of the CICDDoS-2019 dataset is the recall. This proves how the threshold approach is responsible for reducing false negative results and accurately identifying DDoS attacks. The model proves to be strong in a benchmark, namely in the F1-score element, which holds the balance between the two elements. The CNN-MLP model built with the Optimizer to the CICDDoS-2019 and InSDN datasets achieved a high F1-score of 0.994% and 0.9998, respectively. Results shown in Figs 7–10.

These outcomes indicate that the Optimizer-equipped CNN-MLP model is consistently good, and often it is the best model compared to the rest on various datasets. The model’s classifier uses a hybrid MLP-CNN architecture to include the best features of both plain networks. It takes advantage of this design to accurately capture complex spatial data. In this way, the incorporation of SHAP-feature selection can fill the necessary holes in terms of features required for classification in Distributed Denial of service attacks detection. Bayesian optimization techniques are used to fine-tune hyperparameters further and thus achieve higher accuracy and efficiency and increased robustness in the dynamic threat environment. Within the hacking model, DDoS protection is offered, and the system’s dynamism in adapting to new threats at runtime is used to bolster an agile network architecture. The research findings demonstrate characteristic features inherent in the suggested method; this kind of method helps a lot in the advancement of the DDoS detection technique. Here are the summary plots of INSDN and CICDDOS2019 datasets with features and impact on model output.

Using the SHAP summary plot shown in Table 1 from the InSDN dataset, this paper identifies the most significant features that impact a machine learning model’s prediction. Those variables with the highest positive impact include Fwd Pkt Len Mean (+ 1.67) which has a very high positive effect on the model’s output, followed by Pkt Size Avg (+ 1.38) and Fwd Seg Size Avg (+ 0.83). On the other hand, Dst Port (-1.44) and Bwd Header Len (-0.27) decrease the predictions, which means they help in lowering the model’s output. Other features like Subflow Fwd Bytes (+0.59) and Flow Duration (+0.26) also have positive effects, while features such as Tot Fwd Pkts (-0.11) and Pkt Len Var (-0.06) have negative effects. The plot highlights the importance of packet length, size, and flow characteristics in the model’s predictive ability when us-ing the InSDN dataset.

**Table 1:** Summary plot for INSDN dataset.

Feature	Impact on Model Output (f(x))
**Fwd Pkt Len Mean**	+1.67
**Dst Port**	-1.44
**Pkt Size Avg**	+1.38
**Pkt Len Mean**	+0.98
**Fwd Seg Size Avg**	+0.83
**Subflow Fwd Byts**	+0.59
**Fwd Act Data Pkts**	+0.32
**Bwd Header Len**	-0.27
**Flow Duration**	+0.26
**Flow IAT Max**	+0.18
**Fwd Pkt Len Std**	+0.12
**Tot Fwd Pkts**	-0.11
**Subflow Fwd Pkts**	-0.1
**Tot Bwd Pkts**	+0.07
**Bwd IAT Mean**	+0.07
**Pkt Len Var**	-0.06
**Init Bwd Win Byts**	-0.05
**Idle Min**	-0.04
**Idle Mean**	+0.03
**21 other features**	-0.11
**Expected Value (E(f(x)))**	-9.53
**Model Output Value (f(x))**	-5.225

The SHAP summary plot Table 2 of the CICDDoS2019 dataset, which is used in this research, describes the importance of the features in a machine learning algorithm, highlighting the positive or negative effect on the model prediction. Some important features like MaxPacketLength (+2.79) and FwdPacketLengthMax (+1.32) enhance the predictive factors, stressing the importance of packet size and length. On the other hand, BwdIATMax (-1.31) and Inbound (-1.01) reduce the prediction accuracy as they are relevant to the backward packet intervals and the inbound traffic characteristics. In summary, the plot emphasizes that packet size, length, and flow characteristics are instrumental in the model’s predictive capability and assist in comprehending and identifying patterns in the data set.

**Table 2:** Summary plot for CICDDoS2019 dataset.

Feature	Impact on Model Output (f(x))
**MaxPacketLength**	+2.79
**FwdPacketLengthMax**	+1.32
**-**	-1.31
**BwdIATMax**	+1.06
**AveragePacketSize**	+1.04
**Inbound**	-1.01
**MinPacketLength**	+1.01
**TotalLengthofFwdPackets**	+0.98
**PacketLengthMean**	+0.80
**AvgFwdSegmentSize**	+0.65
**SubflowFwdBytes**	+0.61
**FwdPacketLengthMin**	+0.59
**FwdIATTotal**	-0.51
**Protocol**	+0.48
**BwdPackets/s**	+0.43
**FwdPacketLengthMean**	+0.29
**min_seg_size_forward**	+0.21
**URGFlagCount**	-0.2
**FlowDuration**	+0.19
**21 other features**	-0.46
**Expected Value (E(f(x)))**	-15.887
**Model Output Value (f(x))**	-6.926

**Fig 11:**
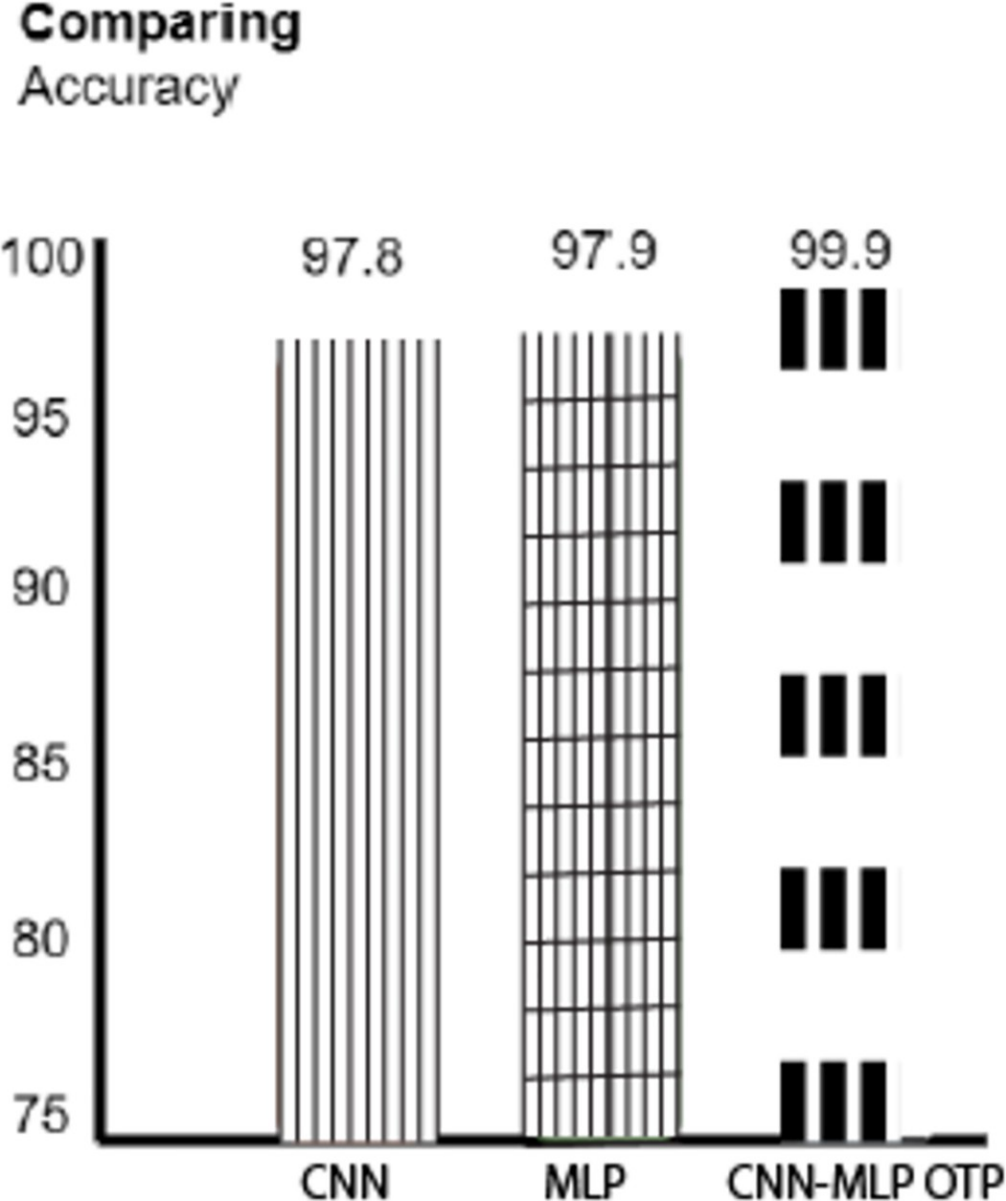
Comparing accuracy.

We also probed into CNN’s ability to detect DDoS attacks in SDN network models. Based on widespread trials with the dataset named "CICDDoS-2019," it was discovered that CNN is an efficient tool that can extract highly complex features in the amount of network traffic. Thus, the SDN infrastructure is more resistant to such attacks because it is much simpler to distinguish between normal and abnormal behavior. Also, MLP has emerged as a very efficient tool for SDN defense in DDoS operations. Having investigated and concluded, the InSDN and CICDDoS-2019 datasets gave amazingly high accuracies. The outcomes of this research demonstrate that MLP is versatile and strong at identifying fraudulent activities in different datasets. Fig 11 depicts the match precision numbers.

An elaborate examination of the MLP and CNN techniques reveals each technique’s main pros and cons. Both models prove to have a good level of accuracy for this task, although LMP is somewhat superior, especially when the results of the InSDN dataset are considered. This result highlights the centrality of the dataset features in deciding how well the machine learning algorithms accurately detect the outflow control inside the SDN system. The bar chart Fig 11 provides a visual representation of the accuracy comparison among three neural network models: CNN, MLP, and CNN-MLP OTP, which are acronyms for memorialized and recorded information. Here is a detailed analysis: Here is a detailed analysis:

1. CNN (Convolutional Neural Network): CNNs are widely used in image recognition since they can apply feature hierarchies from the image inputs automatically and dynamically. This indicated that the CNN model has an accuracy of 97%. Below is how Merge Farms is utilizing deep learning to excel in feature extraction and recognition required for the given task, as indicated by 8%.
2. MLP (Multilayer Perceptron): Feedforward artificial neural networks, often referred to as MLPs, encompass multiple layers of nodes, also known as nodes fully connected. The only exception is input nodes; all other nodes use a nonlinear activation function. The MLP model has a slightly higher accuracy than the CNN, with a 97% accuracy rate. 9%. This small margin indicates that although MLPs are also effective, they do not necessarily represent the spatial details as well as CNNs. However, they perform almost equally well because of the nature of the tasks.
3. CNN-MLP with OPT (Optimal Transport Problem): Using the CNN and MLP models with OPT-eligible method results in a better accuracy level of 99.9%. This model likely benefits from the strengths of both architectures: the CNN that excels at space information processing and the MLP that is more effective at abstract data processing. The OPT aspect might point to another highly advanced optimization procedure that makes the model even more efficient by decreasing some cost functions. This increase in accuracy means that this hybrid model exploits all the peculiarities of the CNN and takes the best result from the MLP, optimizing it and adding further layers of efficiency.

Therefore, all these analyses point out that while both CNN and MLP are very capable models on their own, the enhancement, addition, and overall integration of these models in an optimization way (as in CNN-MLP OTP) leads to a model with even better performance than the individual models. Owing to the increase in the number of dealt operations, accuracy improved to a range of 99%. 9 percent to highlight the possibility of combining hybrid models and optimization methodologies to obtain high accuracy rates for various complex tasks requiring spatial and abstractions. From this flow, the conclusion can be made that for comparable projects, joining the traditional and new methods can be effective if optimal use is made of the possibility that these provide.

In this paper, the utilization of MLP and CNN models combined with SDN provides benefits and drawbacks in aspects such as network latency and performance. Especially during peak traffic time or if the network is under some form of a DDoS attack. For particular scenarios, the system is designed to provide near real-time analysis of the traffic data and work in conjunction with other regular forwarding operations. This is attained through flow design where the SDN controller uses a selective manner taking samples of traffic data and passing it to the model without necessarily affecting normal network traffic. This approach helps ensure that under typical network circumstances, users should not feel that their networks’ performance is severely impacted by our security model. However, high-traffic scenarios are a little more involved And more than one not, due to several reasons. This hostile traffic, coupled with an overload of legitimate traffic, especially when the network is under a DDoS attack, might also lead to increased response time required for our model to detect the attack. To overcome this problem, we have used an adaptive sampling rate as briefly discussed above. This system does not tend to fix the rate and number of traffic sample frequencies constantly with time but rather adapts to the prevailing network traffic. Also, to enhance high availability, we have implemented a function that prioritizes traffic based on the probability of it being malicious and preserves system performance for essential patterns while examining significant volumes of traffic.

The efficacy of the model during attack scenarios is vital, for this is when they are required most. Our proposed hybrid MLP-CNN is a model for feature extraction and rending classification that makes it possible to make the detection faster especially during high-stress situations which can be expected during a DDoS attack. The MLP component enables rapid preliminary examination of the traffic pattern and the CNN is well suited for depicting more intricate spatial characteristics of the traffic that may signify an on-going attack. The SDN controller is also able to provide an immediate counteraction as soon as an attack is recognized. This rapid response capability might also decrease the level of impact on the network as it identifies and eliminates malicious traffic that can flood the network with excessive traffic. To increase performance even more and minimize their effect on network latency, several advanced procedures have been used. Batch processing also enables the model to handle a large number of traffic samples at once resulting in more throughput. Model parallelization imposes the load proportionally to different processors to reduce the time taken for analysis. We also encourage the use of hardware acceleration like a Graphics Processing Unit (GPU) that reduces the processing time. Such optimizations are interdependent in the sense that they help to reduce the effect on the overall network delay and at the same time improve the level of accuracy of threat identification. In case of long-term monitoring but no high-frequency full-scale analysis, we apply the sliding windowing strategy. This approach is useful for updating the network conditions by using the recent traffic data to define the overall trends. It does this without significantly increasing computational overhead but must be able to detect such changes in patterns of attack.

Another important factor which is size, is critical because some networks may be very large. In response to this challenge, we suggest a hierarchical deployment model. In this configuration, local controllers are responsible for preliminary traffic analysis; for this, simplified models are used that allow for approximately, at the first stage, identifying threats. However, when the system detects some patterns, it raises the alarm and sends the findings to a central controller. This centralized controller then applies our full MLP-CNN for further analysis on the data gathered. I get to keep low latency for the nice traffic across the whole network while doing a good job in analyzing potential threats at the same time.

**Fig 12:**
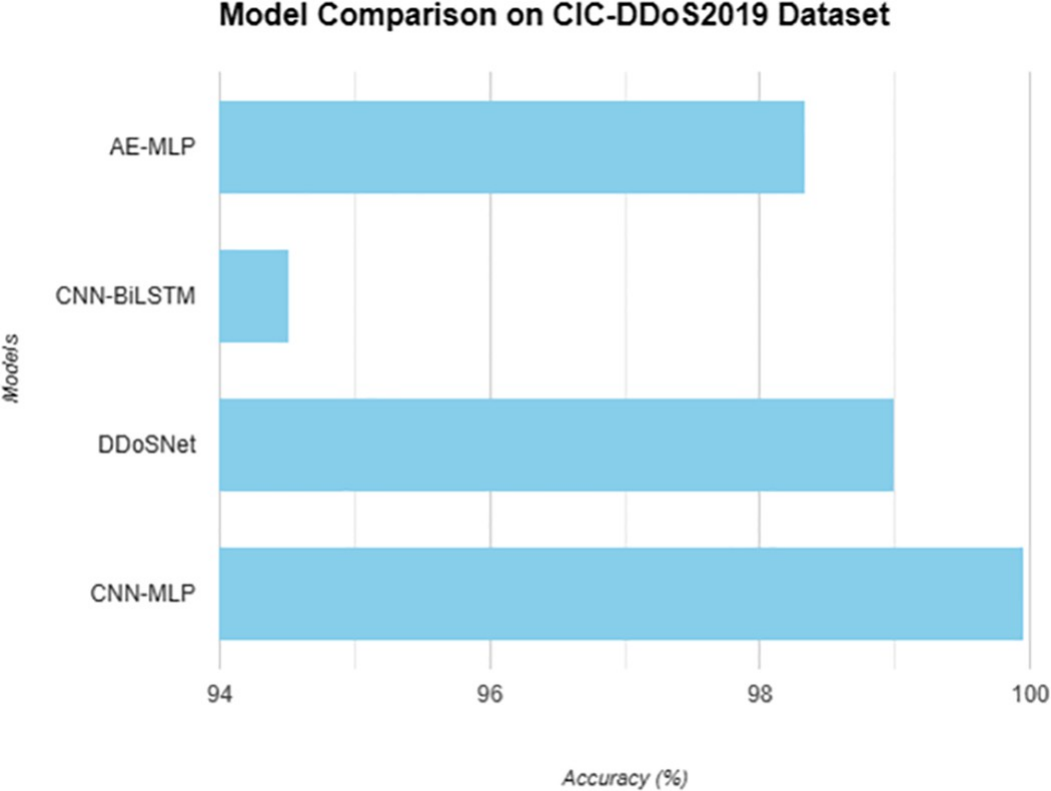
Comparison of models.

Summarizing the results obtained with the proposed models and comparing their performance accuracy in relation to the CIC-DDoS2019 dataset reveals the efficiency of various approaches to the classification and detection of DDoS attacks. This paper provides important insight into the best and worst approaches to employing multiple machine learning architectures to counter one of the most common security threats in current interconnected networks. Through such modeling techniques, including AE-MLP, CNN-BiLSTM, DDosNet, and CNN-MLP with the optimizer, the proficiency of the models can be compared, and their aptITUDe in differentiating between anomalous traffic and normal traffic can be better understood as shown in Fig 12. Therefore, this research becomes important when it comes to determining the appropriate DDoS detection systems to adopt as well as the most suitable methods that would enhance the overall security of the networks. In the following sections of the paper, we review the details of each model and pinpoint its major insights that could be applied to real-life processes in cybersecurity.

The AE-MLP model shows almost perfect results with a recovery rate of over 99% on the CIC-DDoS2019 dataset. This means that the AE-MLP model is well-suited for distinguishing DDoS attacks within this particular data set and, hence, can be employed for DDoS detection. The high accuracy suggests the prototype yields reliable outcomes in detecting normal network traffic from a potential attack. These results may be useful for researchers and practitioners interested in a model with better detection ability, which is applicable, especially for the AE-MLP model. The performance presented in this paper has implications for enhanced protection against DDoS incidents through more accurate prediction models in real-world scenarios.

As for the CNN-BiLSTM model, performance is less advanced compared to the AE-MLP model, as low as below 95% in accuracy. This model still reproduces with relatively high accuracy, but in so doing, it is significantly outperforming these same models on this same set. These results indicate that there may be drawbacks regarding the models’ ability to achieve high accuracy when finding other patterns of DDoS attacks from the given data. Yet, there is still a possibility that the CNN-BiLSTM model can be applied to certain areas and settings with limited computational and training time. However, performance might require additional improvement or examination for better outcomes.

The accuracies of both DDoSNet and the CNN- ML P are quite similar, with the CNN—ML P accuracy slightly higher; both are approximately at 98 and 99 percent respectively. This positions them in between such an AE-MLP model of high accuracy as well as the lower-performing CNN-BiLSTM model. However, these models cannot outperform the AE-MLP model to an extremely large extent, but they still provide a relatively high detection rate on the CIC-DDoS2019 data set. Thus, their performance is higher than the performance of the CNN-BiLSTM model, which led to the conclusion about the expediency of using the proposed methods in practical conditions for DDoS attack detection. Depending on the requirements of the fitted system, factors such as model sophistication, interpretation, or deployment convenience may determine the preference between the DDosNet and CNN-MLP.

The percentages of accuracy reported in these models could differ in real numbers. Still, small numbers can also mean critical results in the case of real-world DDoS detection and mitigation. Thus, a more accurate model, for instance, the AE-MLP, used in alerting potential DDos threats might mean early identification of such threats, thus reducing any losses caused by downtime due to such attacks. On the other hand, a not-very accurate model could mean that some false alarms or events have not been detected; hence, the networks can easily be penetrated. Consequently, depending on the chosen model, questions related to the computing power, training time, and other peculiarities of the model should be carefully considered before the selection to ensure that the chosen model is suitable for the deployment conditions and goals in terms of the resource requirements and estimated results.

Finally, the findings of the CNN-MLP with Optimizer model on CICDDoS-2019 and InSDN datasets provided a desirable result and a reference level of performance for DDoS evaluation. Finally, the position of the nucleus looked like the following with an accuracy of 99%: Based on the CICDDoS-2019 dataset, for distinguishing normal traffic and DoS attacks, the model achieved an accuracy of 95% and an impressive precision rate of 99. On the InSDN dataset, the model design achieved 98% accuracy, which indicates that the proposed system performs exceptionally well in classifying network traffic. The results indicate how effective the data analysis with Optimizer-equipped CNN-MLP is in helping achieve such high accuracy rates, proving itself to be a strong basis of cybersecurity products for various networks. Furthermore, the model has some strengths; for instance, it can avoid having many false positives, being 99 percent precise. The classification achieved 99% accuracy on the InSDN dataset, which reinforces the applicability of this algorithm for differentiating between non-attack and attack traffic patterns. Therefore, the results of the current study underscore the CNN-MLP with Optimizer model as a potent strategy that can be used to enhance network security and reduce the risk of DDoS attack threats.

## 4. Conclusion

Our study introduces a novel technique to enhance the effectiveness of ML-based DDoS detection systems by joining the MLP (Multi-layered Perceptron) and CNN (Convolutional Neural Network) methods. With the collaboration of a Bayesian optimizer and the Shapley Additive feature-selection strategy, our model demonstrates a superior overall accuracy rate, having values of 99.95% (0.9995) and 99.98% (0.9998) on the CICDDoS-2019 and InSDN datasets correspondingly. The results prove that our proposed model is better than the existing ones, and it is very successful in detecting DDoS attacks in SDN systems. Therefore, this study offers viable ways for future developments in network security, particularly regarding SDN, and it is an important contribution to the swiftly advancing field of DDoS detection.
